# 1α,25-(OH)2-D3 promotes osteoclast-precursor autophagy and osteoclast differentiation by promoting BECN1 transcription

**DOI:** 10.1590/1678-4685-GMB-2025-0284

**Published:** 2026-10-23

**Authors:** Xi Li, Hui Liu, Yunrong Zhu

**Affiliations:** ¹ Fuzhou Second General Hospital, Department of Orthopedics, Fuzhou, China.; ² Affiliated Jiangyin Hospital of Xuzhou Medical University, Department of Orthopedics, Jiangyin, China.

**Keywords:** 1α,25-(OH)_2_-D_3_, osteoclast, autophagy, BECN1, p65

## Abstract

1α,25-(OH)_2_-D_3_ can promote autophagy of osteoclast precursors (OCPs), resulting in its pro-osteoclastogenic effect, which limits its anti-osteoporotic efficacy. To address the above challenges, we need to explore the targets of autophagy-enhancing effect of 1α,25-(OH)_2_-D_3_. These results showed that 1α,25-(OH)_2_-D_3_ promoted the expression of BECN1 mRNA and Beclin1 protein in OCPs under RANKL induction. Furthermore, 1α,25-(OH)_2_-D_3_ enhances p65 phosphorylation and nuclear translocation. The lentiviral-mediated silencing of both BECN1 and p65 impeded the facilitation of 1α,25-(OH)_2_-D_3_ on osteoclast differentiation and OCP autophagy. Moreover, p65 silencing blocked the enhancement of BECN1 mRNA expression and Beclin1 protein expression by 1α,25-(OH)_2_-D_3_. In conclusion, 1α,25-(OH)_2_-D_3_ enhances Beclin1-dependent autophagy in OCPs by promoting BECN1 transcription, thereby promoting osteoclast differentiation; this indicates that the therapeutic strategies for BECN1 or p65 targeted inhibition have the potential to optimize the anti-osteoporotic function of 1α,25-(OH)_2_-D_3_.

1α,25-(OH)_2_-D_3_ is a pivotal regulator of bone homeostasis and a core therapeutic agent for osteoporosis treatment (LeBoff et al., 2022; Morin et al., 2025). Nevertheless, accumulating evidence has demonstrated that 1α,25-(OH)_2_-D_3_ facilitates osteoclast differentiation (Sato et al., 2007; Gu et al., 2015; Im et al., 2016), which compromises its anti-osteoporotic efficacy. In this context, targeting the pro-osteoclastogenic effect of 1α,25-(OH)_2_-D_3_ to optimize its therapeutic performance against osteoporosis is of great research value. Mechanistically, prior studies have verified that 1α,25-(OH)_2_-D_3_ induces autophagy in osteoclast precursor cells (OCPs) during osteoclastogenesis, which serves as a critical mechanism underlying its pro-osteoclastogenic activity (Ji et al., 2019; Yu et al., 2021). Therefore, inhibiting 1α,25-(OH)_2_-D_3_-mediated OCP autophagy is the key strategy to improve its anti-osteoporotic function, which necessitates the identification of crucial molecular targets involved in 1α,25-(OH)_2_-D_3_-regulated OCP autophagy.

As the main autophagy regulatory molecule, Beclin1 promotes autophagic responses by activating type-III phosphoinositide 3-kinase (PI3KC3) (Furuya et al., 2005; Pattingre et al., 2005). As a core inducer, RANKL drives osteoclast differentiation through multiple downstream signaling pathways (Boyle et al., 2003). During RANKL-induced osteoclast differentiation, the transcriptional activation of BECN1, subsequent functional expression of Beclin1, and ultimate initiation of autophagy have been well documented (Xiu et al., 2014; Laha et al., 2019; Cai et al., 2023). As a core transcription factor, NF-κB serves as a key regulator governing the transcription of BECN1 (Xiu *et al*., 2014; Cai *et al*., 2023). Our preliminary experimental results confirmed that 1α,25-(OH)_2_-D_3_ upregulates BECN1 mRNA levels in OCPs. Collectively, we hypothesize that 1α,25-(OH)_2_-D_3_ facilitates BECN1 transcription, which elevates Beclin1 expression and activates autophagy in OCPs, ultimately accelerating osteoclast differentiation.

The advantage of our study lies in the first revelation of two important targets of 1α,25-(OH)_2_-D_3_-enhanced OCP autophagy, BECN1 and NF-κB p65, which provides more clues for 1α,25-(OH)_2_-D_3_-treated osteoporosis. Moreover, a previous study demonstrated that the effects of 1α,25-(OH)_2_-D_3_ on OCP autophagy are completely opposite in the presence and absence of RANKL (Ji et al., 2019). Our work demonstrates originality by providing an in-depth molecular mechanism-based exploration for the relationship between 1α,25-(OH)_2_-D_3_ and OCP autophagy under RANKL induction; this is instrumental for elucidating the pharmacological effects of 1α,25-(OH)_2_-D_3_ and refining therapeutic strategies.

Preparation of OCPs and osteoclastic differentiation assay: the tibiae from 4~6-week-old C57BL/6J mice were flushed with alpha-minimum essential medium (α-MEM). After incubation for 24 hours, non-adherent cells from bone marrow cells were collected and incubated on the dishes containing M-CSF (20 ng/ml; Proteintech) for 3 days. The collected adherent cells were considered bone marrow-derived macrophages (BMMs) and used as OCPs. OCPs were cultured in a humidified environment of 37 ℃ and 5% CO_2_. OCPs (6000 cells/well) were incubated in 96-well plates in α-MEM with M-CSF plus RANKL (40 ng/ml; Proteintech) for 4 days to induce osteoclasts. Osteoclastogenic capacity was evaluated using a TRAP staining kit (Sigma-Aldrich) according to the manufacturer’s protocols. The specific procedure is as follows: after fixed and permeabilized, cells were incubated with freshly prepared TRAP staining working solution at 37 °C constant temperature for 30-40 min in the dark humidified environment. The staining reaction was terminated according to the color development observation, and the cells were rinsed carefully with double-distilled water to remove residual staining solution. Subsequently, nuclear counterstaining was performed lightly, and the cells were air-dried naturally. Finally, the stained cells were photographed and observed under an optical microscope for the quantitative analysis of TRAP-positive multinucleated osteoclasts. TRAP-positive cells over 3 nuclei were considered mature osteoclasts. TRAP-positive cells over 5 nuclei were considered large osteoclasts.

Lentiviral Transduction: recombinant lentiviruses encoding shRNA against BECN1/p65 were constructed by homologous recombination between pEX-Puro-Lv105 expression vector and cDNA in 293 cells (CRL-3216; ATCC, Manassas, VA, USA) using the relevant construction kit (GeneCopoeia). When the supernatants were collected after 3 days, OCPs were incubated in medium containing lentiviruses and 5 μg/ml polybrene at a multiplicity of infection (MOI) of 30 for 2 d. The infected cells were selected by puromycin (7.5 μg/ml). The silencing efficiency of shRNAs-lentiviruses was assessed through Western-Blot assay.

Western-Blot assay: the whole cell lysates were prepared, packaged into 10% Sodium dodecyl sulfate polyacrylamide gel (SDS-PAGE) and transferred to polyvinylidene fluoride membranes (PVDF). The PVDFs were incubated with antibodies targeting Beclin1 (11306-1-AP, 1:1000), LC3 (14600-1-AP, 1:5000), p65 (66535-1-Ig, 1:1000), phosphorylated p65 (p-p65; 82335-1-RR, 1:5000), Lamin B1 (12987-1-AP, 1:20000), GAPDH (1:100000) (Proteintech, Wuhan, China) and F-actin (bs-1571R, 1:4000) (Bioss, Beijing, China). Subsequently, PVDFs were incubated with horseradish peroxidase (HRP)-linked secondary antibody to display protein signals. The signals were visualized by using an ECL kit (Millipore, MA, USA).

Quantitative real-time PCR (qRT-PCR) assay: the total RNA from OCPs was extracted and purified by Trizol methods. cDNA synthesis and qRT-PCR assay were performed according to the manufacturer’s protocols (Takara, Tokyo, Japan). The sequences of designed primers are following: *BECN1-*Forward (5’-3’): CTGGAGTCAGAGCTCGATATTT; *BECN1-*Reverse (5’-3’): GACAAGTTGCATGTAGTTGTGT; *p65-*Forward (5’-3’): ATGGCAGACGATCTGTTTCCC; *p65-*Reverse (5’-3’): GGGTGCGTCTTGTTGTTCTT; *Cyclophillin A-*Forward (5’-3’): CGAGCTCTGAGCACTGGAGA; *Cyclophillin A-*Reverse (5’-3’): TGGCGTGTAAAGTCACCACC. During the qRT-PCR assay, the melting curves were analyzed to avoid amplification artifacts. qRT-PCR was performed using SYBR Premix Ex TaqTM kit (TakaRa, Tokyo, Japan) and ABI7500 PCR system (Applied Biosystems, ThermoFisher Scientific, MA, USA).

Nuclear and Cytoplasm Separation: the cytoplasmic and nuclear protein was extracted using the relevant extraction kit (Beyotime, Shanghai, China). 200 µL of cytoplasmic protein extraction reagent was added into harvested OCPs, and OCPs were vortexed followed by ice bath for 10 min. Following high-speed centrifugation for 5 min, the supernatant was harvested for obtaining cytoplasmic protein. And then, 50 µL of nuclear protein extraction reagent was added into the precipitate. The nuclear suspension was vortexed followed by ice bath for 30 min. Next, the nuclear suspension was centrifuged to harvest the supernatant, thereby obtaining nuclear protein. Finally, the protein was used for Western-Blot assay.

Transmission Electron Microscopy (TEM) analysis: For ultrastructural observation of autophagosomes/autolysosomes, the indicated OCPs were seeded and cultured in 6-cm culture dishes. After corresponding treatments, cells were harvested and fixed with 2.5% glutaraldehyde solution at 4 °C overnight. Following thorough rinsing, cells were post-fixed with 1% osmium tetroxide, dehydrated in a graded ethanol series, and embedded in epoxy resin. Ultrathin sections were prepared, stained with uranyl acetate and lead citrate in sequence. All procedures for cell embedding, sectioning and staining were strictly performed following the standard protocols provided by Servicebio (Wuhan, Hubei, China). Ultrastructural changes and autophagosome/autolysosome formation in each group were finally observed and imaged under a Hitachi 7700 transmission electron microscope (Tokyo, Japan). The TEM operating accelerating voltage was set to 80 kV. Autophagosome/autolysosome ultrastructure and intracellular morphological changes were observed and imaged at multiple magnifications ranging from 5,000× to 20,000×.

Chromatin immunoprecipitation (ChIP) assays: ChIP assays were performed to assess the binding activity of p65 to the BECN1 promoter. Briefly, cells were cross-linked with 1% formaldehyde at room temperature for 10 min, followed by quenching with 125 mM glycine for 5 min. Cells were then lysed in SDS lysis buffer, and chromatin was sheared into fragments of 200-500 bp using a sonicator (e.g., Bioruptor Pico, Diagenode). The sonicated chromatin was pre-cleared with protein A/G agarose beads and immunoprecipitated overnight at 4 °C with p65 antibody (Cell Signaling Technology, 1:100) or normal rabbit IgG as a negative control. After washing, immunoprecipitated complexes were eluted, and cross-links were reversed at 65 °C overnight. DNA was purified using a QIAquick PCR Purification Kit (Qiagen). Quantitative PCR (qPCR) was performed on the ABI7500 PCR system using primers specific to the NF-κB binding site-containing region of the BECN1 promoter:

Forward: 5’-TGGCTGGTGATGTTGATGGT-3’;

Reverse: 5’-CCTGCTGCTCTTTCCTGTTG-3’.

The relative enrichment of p65 at the BECN1 promoter was calculated as the percentage of input DNA (% Input), where the total input chromatin was defined as 100%.

Immunofluorescence assay: for the immunofluorescence staining related to LC3-puncta formation, the permeabilized OCPs were incubated with LC3 antibody (1:500) at 4 °C overnight, and then stained with ﬂuorochrome-labelled secondary antibody for 1 hour. Finally, cell nuclei were counterstained with DAPI for 15 minutes, and the images were collected using a fluorescent microscopy (Olympus IX81, Tokyo, Japan).

Statistical analyses: the data were expressed as mean±SEM from three independent assays. The statistical analyses were performed using SPSS 26.0 software and One-way ANOVA and Student’s t-test. Bonferroni test was applied for post-hoc multiple comparisons of One-way ANOVA. The differences were considered significant at a threshold of *P*<0.05.

1α,25-(OH)2-D3 promotes BECN1 expression and p65 nuclear translocation in OCPs: we first evaluated the effect of 1α,25-(OH)_2_-D_3_ on BECN1/Beclin1 in OCPs. 1α,25-(OH)_2_-D_3_ increased the number and size of mature osteoclasts in a concentration-dependent manner (The assessment of osteoclast size relies on the measurement of large osteoclast number) as well as F-actin protein expression in OCPs (Figure 1 A, C ), indicating its efficacy in this study. Accordingly, our follow-up results are reliable. Importantly, 1α,25-(OH)_2_-D_3_ enhanced BECN1 mRNA expression in OCPs in a concentration-dependent manner (Figure 1 B ); this opened the test of the above hypothesis. As shown in Figure 1 C , 1α,25-(OH)_2_-D_3_ enhanced the protein expression of Beclin1 and LC3II in OCPs in a concentration-dependent manner; this was consistent with the objective results observed in previous studies (Ji et al., 2019; Yu et al., 2021) and further verified the upregulation of BECN1/Beclin1 by 1α,25-(OH)_2_-D_3_. 1α,25-(OH)_2_-D_3_ increased the number of autophagosomes/autolysosomes (Figure 1 D ), and promoted LC3 conversion (LC3I/II ratio) and LC3-puncta formation in OCPs in the absence or presence of lysosomal protease inhibitors (Figure 1 E, F); this further demonstrated 1α,25-(OH)_2_-D_3_-promoted OCP autophagy and confirmed the reliability of the data related to 1α,25-(OH)_2_-D_3_ we used. LC3 conversion and LC3-puncta formation were more significant under the intervention of lysosomal protease inhibitors in the absence or presence of 1α,25-(OH)_2_-D_3_ (Figure 1 E, F ), confirming the stability of autophagy flux; this further proved the validity of the above data. Notably, 1α,25-(OH)_2_-D_3_ decreased p65 phosphorylation in OCPs in a concentration-dependent manner (Figure 1 C ). ChIP-qPCR results demonstrated that 1α,25-(OH)₂D₃ significantly enhanced p65 enrichment at the NF-κB binding motif-containing region of the BECN1 promoter while IgG controls remained at low background levels (Figure 1 G ), confirming direct p65-mediated transcriptional regulation of BECN1 and the promoting effect of 1α,25-(OH)₂D₃ on p65 enrichment. Furthermore, 1α,25-(OH)_2_-D_3_ increased p65 expression in the nucleus and decreased p65 expression in the cytoplasm (Figure 1 H). The contribution of 1α,25-(OH)_2_-D_3_ to NF-κB p65 activation/nuclear transfer and subsequent BECN1 transcription was suggested.

**Figure 1 -:**
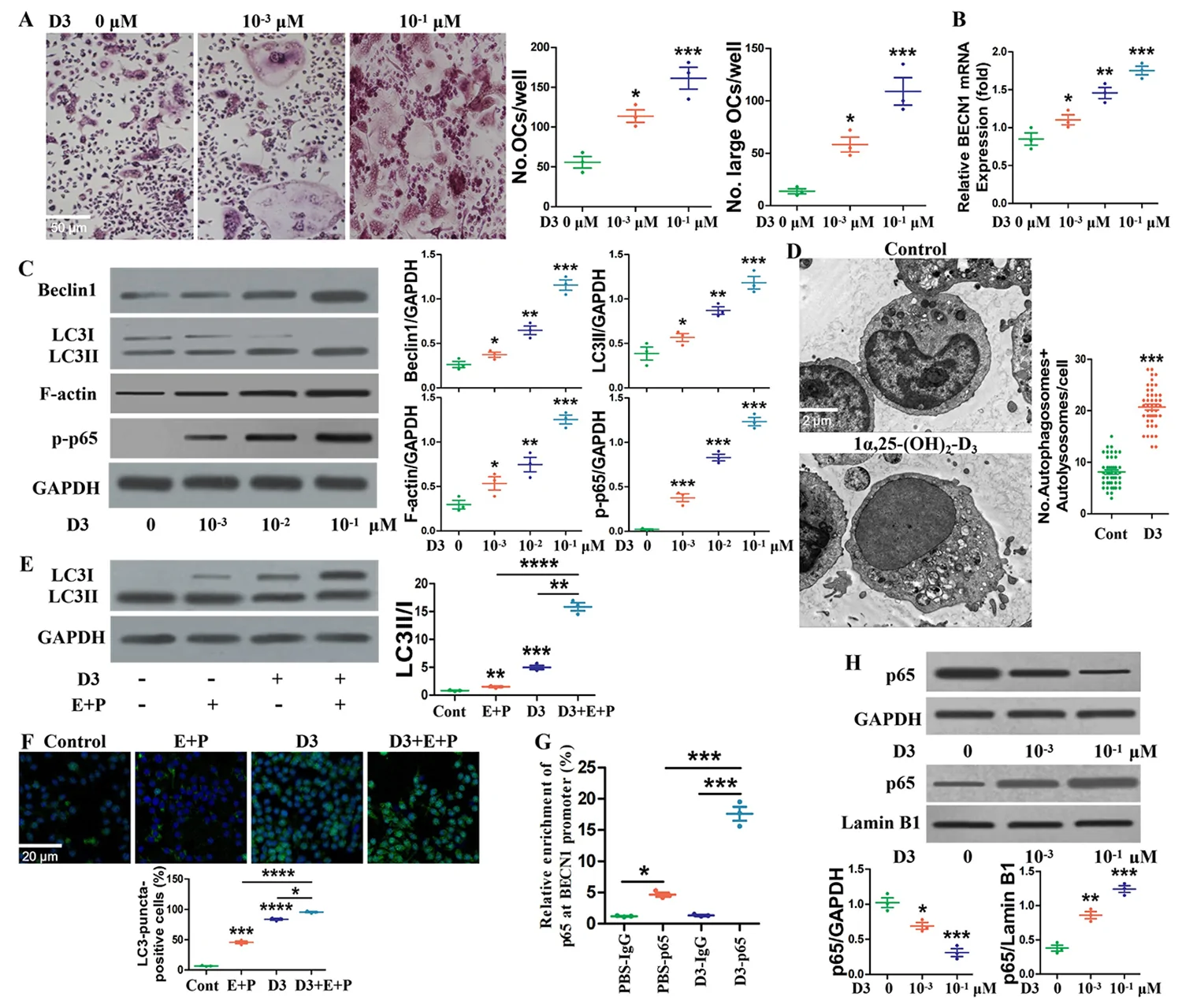
1α,25-(OH)_2_-D_3_ promotes BECN1 expression and p65 nuclear translocation in OCPs. (A) After treatment with different levels of 1α,25-(OH)_2_-D_3_ (0, 10^-3^ and 10^-1^ nM) for 4 days in the presence of RANKL (40 ng/ml) and M-CSF (20 ng/ml), osteoclast differentiation (i.e. TRAP^+^ multinucleate cells) was evaluated by TRAP staining. The histograms represent the quantitative results of differentiated osteoclasts and large osteoclasts with more than 5 nuclei. (B) After treatment with different levels of 1α,25-(OH)_2_-D_3_ (0, 10^-3^, 10^-2^ and 10^-1^ nM) for 8 hours in the presence of RANKL, mRNA levels of BECN1 were detected by qRT-PCR. (C) After treatment with different levels of 1α,25-(OH)_2_-D_3_ for 8 hours in the presence of RANKL, protein levels of Beclin1, LC3, F-actin and p-p65 were detected by Western Blot. LC3 detection focuses on the quantitative results of LC3II. (D) After treatment with 10^-1^ nM of 1α,25-(OH)_2_-D_3_ for 24 hours in the presence of RANKL, the number of autophagosomes/autolysosomes per cell were detected by TEM (a total of 45 cells observed). (E) After treatment with 10^-1^ nM of 1α,25-(OH)_2_-D_3_ for 8 hours in the presence of RANKL along with or without 10 μM E64d plus 10 μM Pepstatin A, protein levels of Beclin1, LC3, F-actin and p-p65 were detected by Western Blot. LC3 detection focuses on LC3II/LC3I ratio. (F) After treatment with 10^-1^ nM of 1α,25-(OH)_2_-D_3_ for 8 hours in the presence of RANKL along with or without E64d plus Pepstatin A, the formation of LC3-puncta were evaluated by immunofluorescence assay. Green punctate fluorescences represent LC3-puncta while blue fluorescences represent DAPI. The cells containing more than 5 LC3-puncta were defined as positive cells. The histograms represent the quantitative results of LC3-puncta-positive cells (Percentage from 250 cells). (G) ChIP-qPCR assays showing the binding activity of p65 to the BECN1 promoter in OCPs treated with PBS or 1α,25-(OH)₂-D₃ for 8 h in the presence of RANKL. IgG was used as a negative control for non-specific binding. The relative enrichment of p65 at the BECN1 promoter is presented as the percentage of input DNA (% Input). (H) After treatment with different levels of 1α,25-(OH)_2_-D_3_ for 8 hours in the presence of RANKL, p65 protein levels in cytoplasm and nucleus were detected by Western Blot. Nuclear quantification depends on Lamin B1 as an internal reference. The data are expressed as the mean±SEM from three independent experiments. ^*^ *P*<0.05, ^**^ *P*<0.01, ^***^ *P*<0.001, ^****^ *P*<0.0001 by One-way ANOVA or Student’s t-test. D3, 1α,25-(OH)_2_-D_3_; Cont, control group; E, E64d; P, Pepstatin A; D3-IgG: 1α,25-(OH)₂D₃-treated cells, ChIP with IgG; D3-p65: 1α,25-(OH)₂D₃-treated cells, ChIP with anti-p65.

BECN1 silencing blocks the effects of 1α,25-(OH)_2_-D_3_: the effects of 1α,25-(OH)_2_-D_3_ on BECN1 expression and OCP autophagy are known. The role of BECN1 in OCP parameters regulated by 1α,25-(OH)_2_-D_3_ also requires clarification. We discovered that BECN1 silencing not only inhibited LC3 conversion and LC3-puncta formation in OCPs, but also blocked the promotion of 1α,25-(OH)_2_-D_3_ on these parameters (Figure 2 A-C ); this confirmed that BECN1 is involved in 1α,25-(OH)_2_-D_3_-regulated OCP autophagy. Additionally, BECN1 silencing not only inhibited F-actin protein expression and the number and size of mature osteoclasts, but also blocked the promotion of 1α,25-(OH)_2_-D_3_ on these parameters (Figure 2 B, D ); this demonstrated that Beclin1-dependent autophagy participates in 1α,25-(OH)_2_-D_3_-regulated osteoclast differentiation.

**Figure 2 -:**
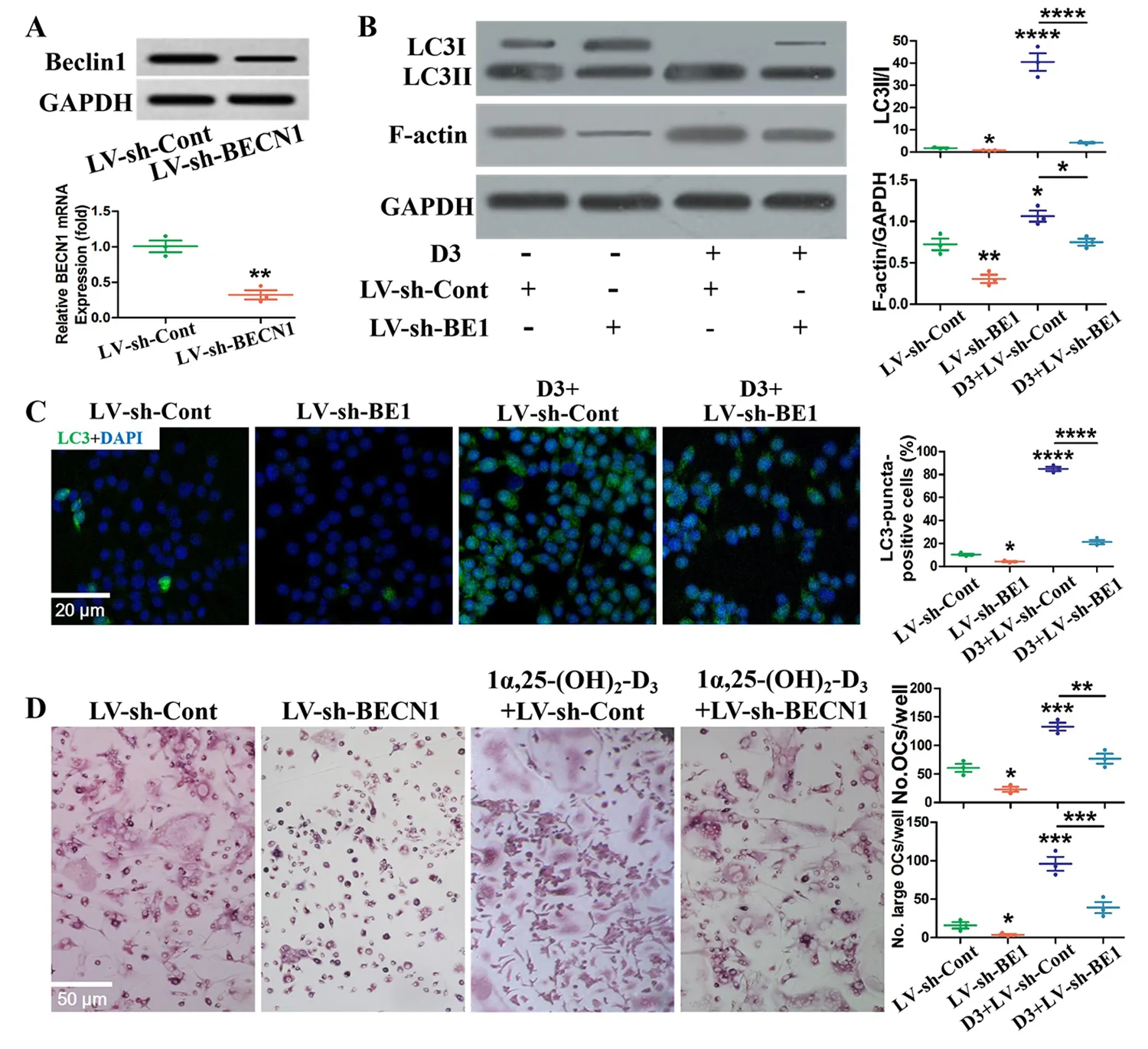
BECN1 silencing blocks the effects of 1α,25-(OH)_2_-D_3._ (A) Beclin1 protein and mRNA expression identifying the lentiviral silencing efficiency of BECN1. (B) After treatment with 10^-1^ nM of 1α,25-(OH)_2_-D_3_ for 8 hours in the presence of RANKL under lentiviral infection, protein levels of LC3 and F-actin were detected by Western Blot. LC3 detection focuses on LC3II/LC3I ratio. (C) After treatment with 10^-1^ nM of 1α,25-(OH)_2_-D_3_ for 8 hours in the presence of RANKL under lentiviral infection, LC3-puncta formation were evaluated by immunofluorescence assay. Green punctate fluorescences represent LC3-puncta while blue fluorescences represent DAPI. The cells containing more than 5 LC3-puncta were defined as positive cells. The histograms represent the quantitative results of LC3-puncta-positive cells (Percentage from 250 cells). (D) After treatment with 10^-1^ nM of 1α,25-(OH)_2_-D_3_ for 4 days in the presence of RANKL and M-CSF under lentiviral infection, osteoclast differentiation was evaluated by TRAP staining. The histograms represent the quantitative results of differentiated osteoclasts and large osteoclasts with more than 5 nuclei. The data are expressed as the mean±SEM from three independent experiments. ^*^ *P*<0.05, ^**^ *P*<0.01, ^***^ *P*<0.001, ^****^ *P*<0.0001 by One-way ANOVA. D3, 1α,25-(OH)_2_-D_3_; LV-sh-Cont, control shRNA lentivirus; LV-sh-BE1, BECN1 shRNA lentivirus.

p65 silencing blocks the effects of 1α,25-(OH)_2_-D_3_: finally, the role of p65 in the OCP parameters regulated by 1α,25-(OH)_2_-D_3_ also needs to be clarified. We discovered that p65 silencing not only inhibited BECN1/Beclin1 mRNA and protein expression, LC3 conversion and LC3-puncta formation in OCPs, but also blocked the promotion of 1α,25-(OH)_2_-D_3_ on these parameters (Figure 3 A-D ); this confirmed that p65 is involved in 1α,25-(OH)_2_-D_3_-regulated BECN1 transcription and OCP autophagy. Moreover, p65 silencing not only inhibited F-actin protein expression and the number and size of mature osteoclasts, but also blocked the promotion of 1α,25-(OH)_2_-D_3_ on these parameters (Figure 3 C, E ); this clarified that p65 and its transcriptional activity are involved in osteoclast differentiation regulated by 1α,25-(OH)_2_-D_3_.

**Figure 3 -:**
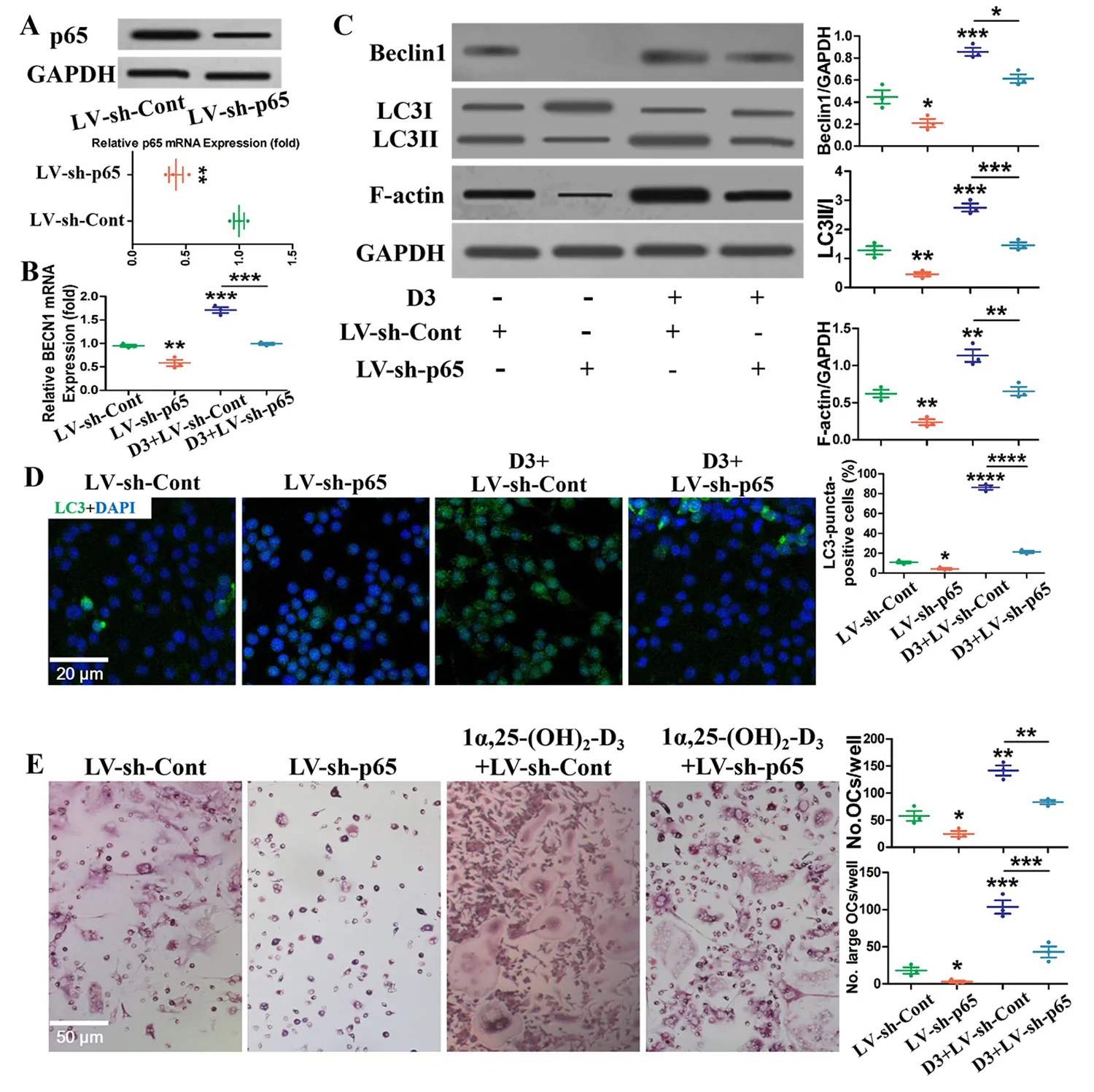
p65 silencing blocks the effects of 1α,25-(OH)_2_-D_3._ (A) p65 protein and mRNA expression identifying the lentiviral silencing efficiency of p65. (B) After treatment with 10^-1^ nM of 1α,25-(OH)_2_-D_3_ for 8 hours in the presence of RANKL under lentiviral infection, BECN1 mRNA levels were detected by qRT-PCR. (C) After treatment with 10^-1^ nM of 1α,25-(OH)_2_-D_3_ for 8 hours in the presence of RANKL under lentiviral infection, protein levels of Beclin1, LC3 and F-actin were detected by Western Blot. LC3 detection focuses on LC3II/LC3I ratio. (D) After treatment with 10^-1^ nM of 1α,25-(OH)_2_-D_3_ for 8 hours in the presence of RANKL under lentiviral infection, LC3-puncta formation were evaluated by immunofluorescence assay. Green punctate fluorescences represent LC3-puncta while blue fluorescences represent DAPI. The cells containing more than 5 LC3-puncta were defined as positive cells. The histograms represent the quantitative results of LC3-puncta-positive cells. (E) After treatment with 10^-1^ nM of 1α,25-(OH)_2_-D_3_ for 4 days in the presence of RANKL and M-CSF under lentiviral infection, osteoclast differentiation was evaluated by TRAP staining. The histograms represent the quantitative results of differentiated osteoclasts and large osteoclasts with more than 5 nuclei. The data are expressed as the mean±SEM from three independent experiments. ^*^ *P*<0.05, ^**^ *P*<0.01, ^***^ *P*<0.001, ^****^ *P*<0.0001 by One-way ANOVA. D3, 1α,25-(OH)_2_-D_3_; LV-sh-Cont, control shRNA lentivirus; LV-sh-p65, p65 shRNA lentivirus.

Current research data show that 1α,25-(OH)₂-D_3_ promotes p65 nuclear translocation by activating the NF-κB signaling pathway, and then upregulates BECN1 expression to promote Beclin1-dependent autophagy, ultimately driving the formation of F-actin and the maturation of OCPs towards osteoclasts. As a classical transcriptional activator, p65 has strong DNA-binding activity, which triggers the transcription of downstream target genes (Lesner et al., 2005; Liu et al., 2008). BECN1, as the gene form of Beclin1, is essential for maintaining effective autophagy flux cycling and autophagy activation in multiple cell types, including osteoclast lineage cells (Xiu et al., 2014; Arai et al., 2019; Laha et al., 2019; Cai et al., 2023; Cao et al., 2024). Furthermore, Beclin1 can promote RANKL-induced osteoclastogenesis by inducing reactive oxygen species production and NFATc1 activation even under non-autophagic conditions (Chung et al., 2014). Accumulated evidence has confirmed that NF-κB signaling facilitates effective BECN1 transcription during RANKL-driven osteoclast differentiation (Xiu *et al*., 2014; Cai *et al*., 2023).

The present study characterizes a regulatory axis linking 1α,25-(OH)₂-D_3_ stimulation, NF-κB p65-mediated BECN1 transcriptional activation, OCP autophagy, and subsequent osteoclast maturation. Although previous studies have separately reported the individual roles of vitamin D, NF-κB signaling, and autophagy in osteoclast biology, the mechanistic connection between 1α,25-(OH)₂-D_3_-triggered p65 transcriptional activity and direct BECN1 gene induction during autophagy-dependent osteoclastogenesis has not been systematically clarified before. Compared with earlier studies focusing mainly on the mainstream osteoclastogenic signaling, our findings further expand the regulatory network of vitamin D in bone metabolism by highlighting the transcriptional regulatory mechanism governing autophagy initiation at the gene level rather than only post-translational protein modification.

Beyond osteoclast lineage regulation, autophagy has been increasingly recognized as a core conserved signaling node controlling the differentiation and function of multiple bone cells. The role of Beclin1-dependent autophagy in osteogenesis has been demonstrated by multiple studies (Wu et al., 2021; Ke et al., 2022; Li et al., 2022), confirming that BECN1/Beclin1-autophagy signaling critically regulates both osteogenic and osteoclastic lineage commitment. Consistent with this concept, BECN1/Beclin1-dependent autophagy axis characterized in our study represents a shared core autophagy machinery across bone cells. Notably, whether the 1α,25-(OH)₂-D_3_-p65-BECN1 regulatory axis exerts distinct, cell-type-specific effects on osteoblasts versus osteoclasts remains worthy of further investigation, as such differential regulatory patterns are essential for balancing bone formation and bone resorption under osteoporosis therapy.

Clinically, 1α,25-(OH)₂-D_3_ is rarely used as a single-agent therapy for osteoporosis, and generally administered in combination with other bone-protective drugs, as monotherapy usually yields minimal therapeutic benefits in patients. All experiments in this study were performed in an *in vitro* OCP culture system. Although the *in vitro* cell model enables precise mechanistic exploration of molecular signaling, it cannot fully recapitulate the complex *in vivo* bone microenvironment. Future *in vivo* validation studies should focus on confirming the regulatory role of the 1α,25-(OH)₂-D_3_-p65-BECN1 axis in bone remodeling under physiological and pathological conditions. Further animal experiments are also needed to explore the optimized combination regimens of 1α,25-(OH)₂-D_3_ with p65 or BECN1 targeted intervention to improve the anti-osteoporotic efficacy. Overall, these findings clarify the molecular mechanism of vitamin D metabolites coordinating OCP autophagy during osteoclast differentiation through NF-κB signaling. The working model of our study is presented in Figure 4.

**Figure 4 -:**
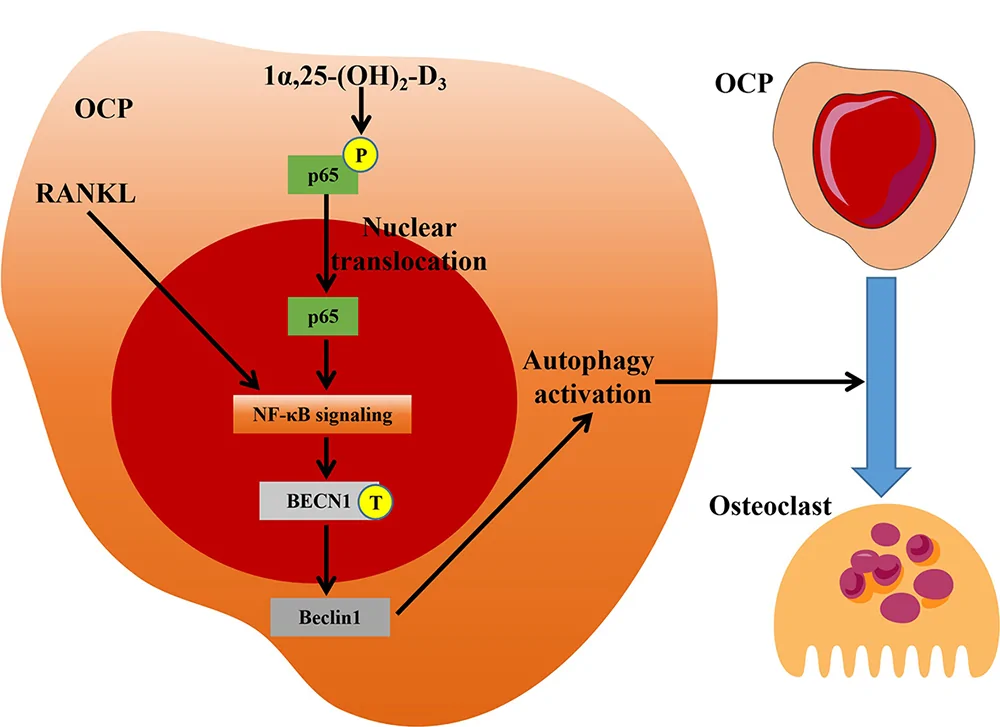
The working model about the promoting effect of 1α,25-(OH)_2_-D_3_ on OCP autophagy and osteoclast differentiation via BECN1 transcription. P, phosphorylation; T, transcription.Abbreviations: OCPs, osteoclast precursors; PI3KC3, type-III phosphoinositide 3-kinase; α-MEM, alfa-minimum essential medium; BMMs, bone marrow-derived macrophages; SDS-PAGE, Sodium dodecyl sulfate polyacrylamide gel; qRT-PCR, Quantitative real-time PCR; PVDF, polyvinylidene fluoride membranes; MOI, multiplicity of infection; PVDF, polyvinylidene fluoride membranes; TEM, Transmission Electron Microscopy; HRP, horseradish peroxidase.

The clinical evidence confirms that 1α,25-(OH)₂-D_3_ is applied as an important anti-osteoporotic drug (LeBoff et al., 2022; Morin et al., 2025). However, the limited efficacy of 1α,25-(OH)₂-D_3_ may be partially attributed to its dual effects on bone metabolism, particularly its potential side effect of promoting excessive osteoclastic bone resorption. Although our *in vitro* findings suggest that targeting p65 or BECN1 may help attenuate the osteoclast-activating effect of 1α,25-(OH)₂-D_3_, such therapeutic speculation remains preliminary and should be interpreted with caution due to the complexity of *in vivo* bone homeostasis and potential off-target effects of long-term gene or pharmacological inhibition. Future rigorous *in vivo* validation and safety evaluation are necessary before translating these molecular targets into clinical optimized anti-osteoporosis strategies.

## Data Availability

The raw data supporting the findings in this study are available upon reasonable request.

## References

[B1] Arai A, Kim S, Goldshteyn V, Kim T, Park NH, Wang CY, Kim RH (2019). Beclin1 modulates bone homeostasis by regulating osteoclast and
chondrocyte differentiation. J Bone Miner Res.

[B2] Boyle WJ, Simonet WS, Lacey DL (2003). Osteoclast differentiation and activation. Nature.

[B3] Cai S, Chen Y, Chen J, Wei W, Pan J, Wu H (2023). Rubiadin-1-methyl ether inhibits BECN1 transcription and
Beclin1-dependent autophagy during osteoclastogenesis by inhibiting NF-κB p65
activation. Exp Biol Med (Maywood).

[B4] Cao Z, Tian K, Ran Y, Zhou H, Zhou L, Ding Y, Tang X (2024). Beclin-1: A therapeutic target at the intersection of autophagy,
immunotherapy, and cancer treatment. Front Immunol.

[B5] Chung YH, Jang Y, Choi B, Song DH, Lee EJ, Kim SM, Song Y, Kang SW, Yoon SY, Chang EJ (2014). Beclin-1 is required for RANKL-induced osteoclast
differentiation. J Cell Physiol.

[B6] Furuya N, Yu J, Byfield M, Pattingre S, Levine B (2005). The evolutionarily conserved domain of Beclin 1 is required for Vps34
binding, autophagy and tumor suppressor function. Autophagy.

[B7] Gu J, Tong XS, Chen GH, Wang D, Chen Y, Yuan Y, Liu XZ, Bian JC, Liu ZP (2015). Effects of 1α,25-(OH)₂D₃ on the formation and activity of osteoclasts
in RAW264.7 cells. J Steroid Biochem Mol Biol.

[B8] Im NK, Lee DS, Lee SR, Jeong GS (2016). Lupeol isolated from Sorbus commixta suppresses 1α,25-(OH)₂D₃-Mediated
osteoclast differentiation and bone loss in vitro and in vivo. J Nat Prod.

[B9] Ji L, Gao J, Kong R, Gao Y, Ji X, Zhao D (2019). Autophagy exerts pivotal roles in regulatory effects of 1α,25-(OH)₂D₃
on the osteoclastogenesis. Biochem Biophys Res Commun.

[B10] Ke D, Wang X, Lin Y, Wei S (2022). Lactoferrin promotes the autophagy activity during osteoblast
formation via BCL2-Beclin1 signaling. Mol Biol Rep.

[B11] Laha D, Deb M, Das H (2019). KLF2 (kruppel-like factor 2 [lung]) regulates osteoclastogenesis by
modulating autophagy. Autophagy.

[B12] LeBoff MS, Chou SH, Ratliff KA, Cook NR, Khurana B, Kim E, Cawthon PM, Bauer DC, Black D, Gallagher JC (2022). Supplemental vitamin D and incident fractures in midlife and older
adults. N Engl J Med.

[B13] Lesner A, Li Y, Nitkiewicz J, Li G, Kartvelishvili A, Kartvelishvili M, Simm M (2005). A soluble factor secreted by an HIV-1-resistant cell line blocks
transcription through inactivating the DNA-binding capacity of the NF-kappa B
p65/p50 dimer. J Immunol.

[B14] Li X, Zhu Y, Lin X, Chen C, Liu H, Shi Y (2022). Beclin1- and Atg13-dependent autophagy activation and morroniside have
synergistic effect on osteoblastogenesis. Exp Biol Med (Maywood).

[B15] Liu J, Yoshida Y, Yamashita U (2008). DNA-binding activity of NF-kappaB and phosphorylation of p65 are
induced by N-acetylcysteine through phosphatidylinositol (PI)
3-kinase. Mol Immunol.

[B16] Morin SN, Leslie WD, Schousboe JT (2025). Osteoporosis: A review. JAMA.

[B17] Pattingre S, Tassa A, Qu X, Garuti R, Liang XH, Mizushima N, Packer M, Schneider MD, Levine B (2005). Bcl-2 antiapoptotic proteins inhibit Beclin 1-dependent
autophagy. Cell.

[B18] Sato M, Nakamichi Y, Nakamura M, Sato N, Ninomiya T, Muto A, Nakamura H, Ozawa H, Iwasaki Y, Kobayashi E (2007). New 19-nor-(20S)-1alpha,25-dihydroxyvitamin D3 analogs strongly
stimulate osteoclast formation both in vivo and in vitro. Bone.

[B19] Wu H, Xue Y, Zhang Y, Wang Y, Hou J (2021). PTH1-34 promotes osteoblast formation through Beclin1-dependent
autophagic activation. J Bone Miner Metab.

[B20] Xiu Y, Xu H, Zhao C, Li J, Morita Y, Yao Z, Xing L, Boyce BF (2014). Chloroquine reduces osteoclastogenesis in murine osteoporosis by
preventing TRAF3 degradation. J Clin Invest.

[B21] Yu C, Zhu Y, Lv X, Wang Y (2021). 1α,25-(OH)₂-D₃ promotes the autophagy during osteoclastogenesis by
enhancing RANKL-RANK-TRAF6 signaling. Vitro Cell Dev Biol Anim.

